# Distribution and evolution of acute interventional ischemic stroke treatment in Germany from 2010 to 2016

**DOI:** 10.1186/s42466-019-0010-8

**Published:** 2019-02-28

**Authors:** Ralph Weber, Jens Eyding, Martin Kitzrow, Dirk Bartig, Christian Weimar, Werner Hacke, Christos Krogias

**Affiliations:** 1 Department of Neurology, Alfried Krupp Krankenhaus Essen and Ruhr University Bochum Germany, Alfried-Krupp-Str. 21, 45131 Essen, Germany; 2 Department of Neurology, Klinikum Dortmund, Beurhausstr. 40, 44137 Dortmund, Germany; 3 Department of Neurology, Agaplesion Bethesda Hospital, Hainstr. 35, 42109 Wuppertal, Germany; 4 drg market, Akeleiweg 7, 49082 Osnabrück, Germany; 5 Department of Neurology, University Duisburg-Essen, Hufelandstraße 55, 45147 Essen, Germany; 6 Senior Professor of Neurology, University of Heidelberg, Im Neuenheimer Feld 400, 69120 Heidelberg, Germany; 7 Department of Neurology, University Hospital St. Josef-Hospital Bochum, Ruhr University Bochum, Gudrunstraße 56, 44791 Bochum, Germany; 8 Northwest-German Stroke Circle e.V., Bochum, Germany

**Keywords:** Stroke, Thrombectomy, Thrombolysis, Health care structure

## Abstract

**Background:**

Mechanical thrombectomy (MT) is a new evidence-based treatment option for large vessel occlusion in the anterior brain circulation. Using comprehensive administrative data from Germany, we analysed the nationwide development of intravenous thrombolysis (IVT) and MT in Germany between 2010 and 2016.

**Methods:**

We considered all documented cases (*n* = 1,515,634) with a main diagnosis of the ICD-10-GM code I63 (ischemic stroke) and identified specific stroke recanalization therapy by using the corresponding Operating and Procedure Key for systemic thrombolysis and mechanical thrombectomy out of the DRG statistics. Regional analyses are based on data from the 413 German administrative districts and cities and the obligatory quality reports of all hospitals. We distinguished between rates of MT related to place of residence of patients and place of treatment.

**Results:**

Coded ischemic strokes increased by 10.2% from 2010 (*n* = 206.688) to 2016 (*n* = 227.687). The rate of IVT increased from 8.9% in 2010 to 14.9% in 2016 and the rate of MT increased from 0.8% in 2010 to 4.7% in 2016 with a strong increase in 2015 and 2016. There was a high regional variability of MT according to place of residence of patients between 0 and 11.2% in 2016 with significant lower treatment rates in rural compared to urban areas (3.8 vs 5.4%). Mean age of patients treated with MT increased from 67.8 years in 2010 to 73.3 years in 2016 and almost reached the mean age of IVT treated patients (74.4 years). The number of hospitals coding MT increased from 91 to 193 from 2010 to 2016, but 80% of all MT procedures were performed in neurointerventional centers with ≥50 procedures/year in 2016.

**Conclusions:**

The rate of IVT in patients with acute ischemic stroke in Germany continues to rise and has reached 14.9% nationwide. The increase of MT is even more pronounced and was triggered by the evidence after publication of the MT randomized trials. There is still a high regional variability with significant lower MT rates in rural areas.

## Introduction

Stroke accounts for the largest proportion of disability-adjusted life year loss and two-thirds of deaths among all neurological disorders in the Global Burden of Disease Study 2015 [1]. In 2015, five randomized trials showed that mechanical thrombectomy (MT) with stent retrievers is effective and safe in patients with large vessel occlusion (LVO) of the anterior brain circulation if performed within 6 h after stroke onset on top of intravenous thrombolysis (IVT) alone [5–9]. MT has become the third evidence-based column of acute stroke therapy besides stroke unit treatment and IVT with recombinant tissue plasminogen activator (rt-PA) [10–13]. It is estimated that between 7 and 15% of all stroke patients could be candidates for MT [14–16].

Whilst most stroke patients in high income countries have access to stroke unit care, the uptake of IVT was relatively slow. Given these limitations of care, the implementation and access to MT may be even more difficult to achieve. Based on administrative data from all acute care hospitals in all 413 cities/regions of Germany, we analysed the nationwide evolution of IVT and MT in hospitalized acute stroke patients from 2010 to 2016 with a special focus on places of residence and places of treatment of these patients. Furthermore, we assessed differences of MT use dependent on regional population density and frequency of treatment in all German treatment sites. Our findings may help to improve nation-wide acute stroke care including MT.

## Methods

Analyses were based upon the statistical evaluation of the German Diagnosis-Related Groups (G-DRG) data from 2010 to 2016 (DRG-statistic, Federal Statistical Office, www.destatis.de) as well as the mandatory structured quality reports of hospitals (according to §137, 3; Social Code Book V of Germany) from the year 2016, enabling the calculation of IVT and MT rates in each hospital and the number of ischemic stroke patients treated in each hospital^1^. We extracted all cases with the main ICD-10 code I63 (ischemic stroke) and calculated population based incidence, mean age and gender. Cases being transferred once or multiple times from one hospital to another either for acute stroke therapy and/or early rehabilitation were censored appropriately to avoid double and multiple coding (exclusion of “discharge key 06”). Therefore, first ever as well as recurrent strokes were included with the exception of early recurrences that occurred during the ongoing hospital treatment phase for the first incidence. In a cross over analysis, the associated stroke recanalization therapy was categorized by using the corresponding Operating and Procedure Key for systemic thrombolysis (OPS code 8–020.8) and mechanical thrombectomy (OPS 8–836.80). Estimation of bridging-IVT rate was done by cross over analysis of lead-DRGs coding for MT-procedures.

In a first step, data were analyzed based upon the patients’ place of residence. Regional analyses were done by data aggregation considering the 413 German administrative districts and cities. To avoid bias, we excluded all cases of foreign or unknown place of residence from regional statistics.

In a second step, analysing the data from the structured quality reports of the hospitals^2^, we calculated the number of IVT and MT cases in each hospital and the number of acute ischemic stroke patients treated in each hospital. Hospitals without neurological departments coding MT procedures were excluded. This was done due to the assumption that these patients were either treated by interventionalists other than (neuro-) radiologists in cooperation with neurologists, or were most probably coded twice, i.e. in a neurointerventional center and afterwards in a geriatric (or neurological) early rehabilitation clinic. In such a scenario, case assignment would be allocated to the hospital with the geriatric/rehabilitation department.

We stratified hospitals by number of MTs performed per year into the following categories: 1–9, 10–34, 35–49, 50–99, 100–199, > 200. These categories were chosen for the following reasons:

• **Seldom MT treatments** and possible false entries (1–9): with less than one treatment per month no regular experience can be presumed.

• **Occasional MT treatments** (10–34 and 35–49): with less than one treatment per week / every two weeks only occasional experience can be presumed according to undergoing discussions on different minimal requirements for procedures per year.

• **Regular MT treatments** (50–99): with more than 1 treatment per week, a regular experience in interventional stroke treatment can be presumed.

• **Frequent MT treatments** (100–199): with 2 to 4 treatments per week, a good experience of several neurointerventionalists can be presumed and the center can be regarded as a training center.

• **High volume MT treatments** (> 200): with more than 4 treatments per week, a very good experience can be presumed and the neuro-interventionalists can be regarded as experts.

We used the Eurostat definition to stratify the 413 German administrative districts and cities into densely populated areas (cities) with a population density over 500 inhabitants per km^2^ and a minimum population of 50.000 inhabitants, intermediate density areas with a population density between 500 and 100 inhabitants per km^2^, and thinly populated (rural) areas with a population density below 100 inhabitants per km^2^.

We searched PubMed for studies in English language up to September 30, 2018 using the terms “ischemic stroke” AND “mechanical thrombectomy” OR “mechanical recanalization” OR “intraarterial recanalization” OR “thromboaspiration” to identify studies investigating nationwide coverage, regional differences and temporal development of mechanical thrombectomy (MT) in acute ischemic stroke patients after the introduction of modern MT systems (stent retrievers and thromboaspiration systems).

For descriptive analyses, results are reported as absolute numbers and mean. Statistical comparison of groups was performed with the Mann-Whitney-U-test. A *p* value of < 0·05 was considered significant. All analyses were performed with Microsoft Excel and IBM SPSS version 21.

## Results

**Fig. 1:**
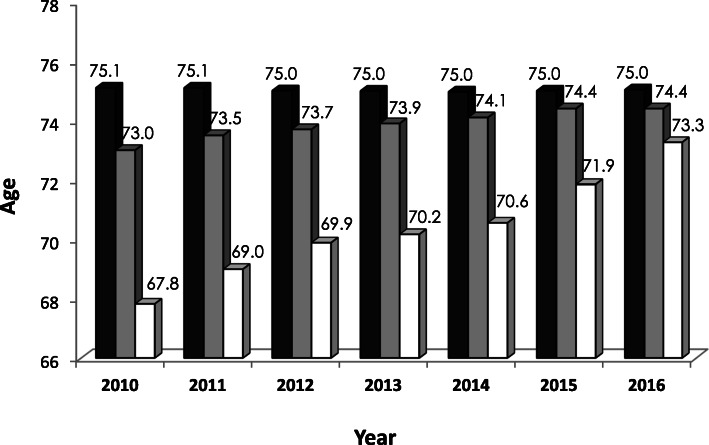
Mean age of all patients with ischemic stroke (black), treated with intravenous (grey) thrombolysis and mechanical thrombectomy (white) in Germany from 2010 to 2016

The administrative hospital data from all German acute care hospitals showed an absolute increase of 10.2% for the total number of ischemic strokes (ICD I63) from 2010 (*n* = 206.688) to 2016 (*n* = 227.687). Mean age of patients with ischemic stroke was 75 years and remained stable over this period, with female stroke patients being significantly older (Table 1 and Fig. 1).

**Table 1:** Rates of ischemic stroke, intravenous thrombolysis and mechanical thrombectomy from 2010 to 2016 in Germany

	2010	2011	2012	2013	2014	2015	2016
Ischemic strokes (ICD I63), n	206.688	209.976	214.157	216.535	218.371	222.220	227.687
Age, mean	75.1	75.1	75.0	75.0	75.0	75.0	75.0
Sex, male (%)	49.0%	49.3%	49.9%	50.4%	50.7%	51.5%	51.8%
IVT overall, n (%)	18.362 (8.9%)	21.381 (10.2%)	24.426 (11.4%)	26.764 (12.4%)	28.447 (13.0%)	30.589 (13.8%)	33.916 (14.9%)
Age, mean	73.0	73.5	73.7	73.9	74.1	74.4	74.4
IVT rate < 80 years, %	10.0%	11.1%	12.2%	13.0%	13.5%	14.1%	15.2%
IVT rate ≥ 80 years, %	7.0%	8.6%	10.1%	11.2%	12.2%	13.1%	14.4%
MT overall, n (%)	1.662 (0.8%)	3.061 (1.5%)	4.505 (2.1%)	5.141 (2.4%)	5.526 (2.5%)	7.840 (3.5%)	10.692 (4.7%)
Age, mean	67.8	69.0	69.9	70.2	70.6	71.9	73.3
MT rate < 80 years, %	1.1%	1.8%	2.6%	2.9%	3.1%	4.0%	5.1%
MT rate ≥ 80 years, %	0.4%	0.8%	1.2%	1.4%	1.6%	2.6%	4.1%

Percentage values are based on all patients with ICD code I63

*ICD* indicates International Statistical Classification of Diseases and Related Health Problems, *IVT* intravenous thrombolysis, *MT* mechanical thrombectomy

The nationwide rate of IVT continuously increased from 8.9% in 2010 to 14.9% in 2016 (Table 1). The IVT rate showed a high variation between German cities and districts in all years analyzed (2016: 2.4–28.0%; Table 1). The mean age of acute stroke patients receiving IVT slightly increased from 73.3 years in 2010 to 74.4 years in 2016 caused by a doubling of the IVT rate in patients over 80 years of age (from 7.0% in 2010 to 14.4% in 2016, Table 1).

**Fig. 2:**
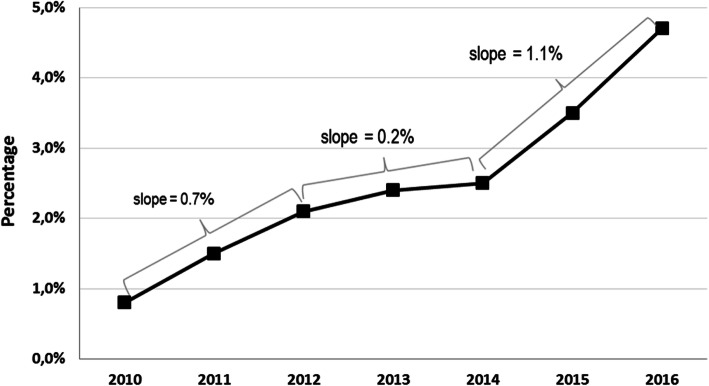
Temporal evolution of mechanical thrombectomy rates in Germany between 2010 and 2016 (Slope is given as absolute percentage points per year)

The overall rate of MT in ischemic stroke patients in Germany increased from 0.8% in 2010 to 4.7% in 2016, with a wide regional range, according to place of residence, between 0 and 11.2% in 2016 (Table 1 and Fig. 2). From 2010 to 2012, the MT rate increased steadily, with a slow-down in 2013 and 2014 (after publication of the first three neutral randomized MT trials in spring 2013), and increased sharply again from 2014 to 2016 (after publication of the positive randomized MT trials) (Table 1 and Fig. 2). In 2016, the mean MT rate in the 20 cities/districts with the highest MT rates was 8.4% (range 7.2 to 11.2%), and the mean MT rate in the 20 cities/districts with the lowest MT rates was 1.0% (range 0 to 1.3%). There was only one small district, in which no ischemic stroke patient received MT in 2016.

The rate of IVT in combination with MT (“bridging approach”) declined from 75% in 2010 to 51% in 2016.

The mean age of patients receiving MT increased from 67.8 years in 2010 to 73.3 years in 2016 and was comparable to the mean age of stroke patients treated with IVT in 2016 (74.4 years; Table 1 and Fig. 1).

**Fig. 3:**
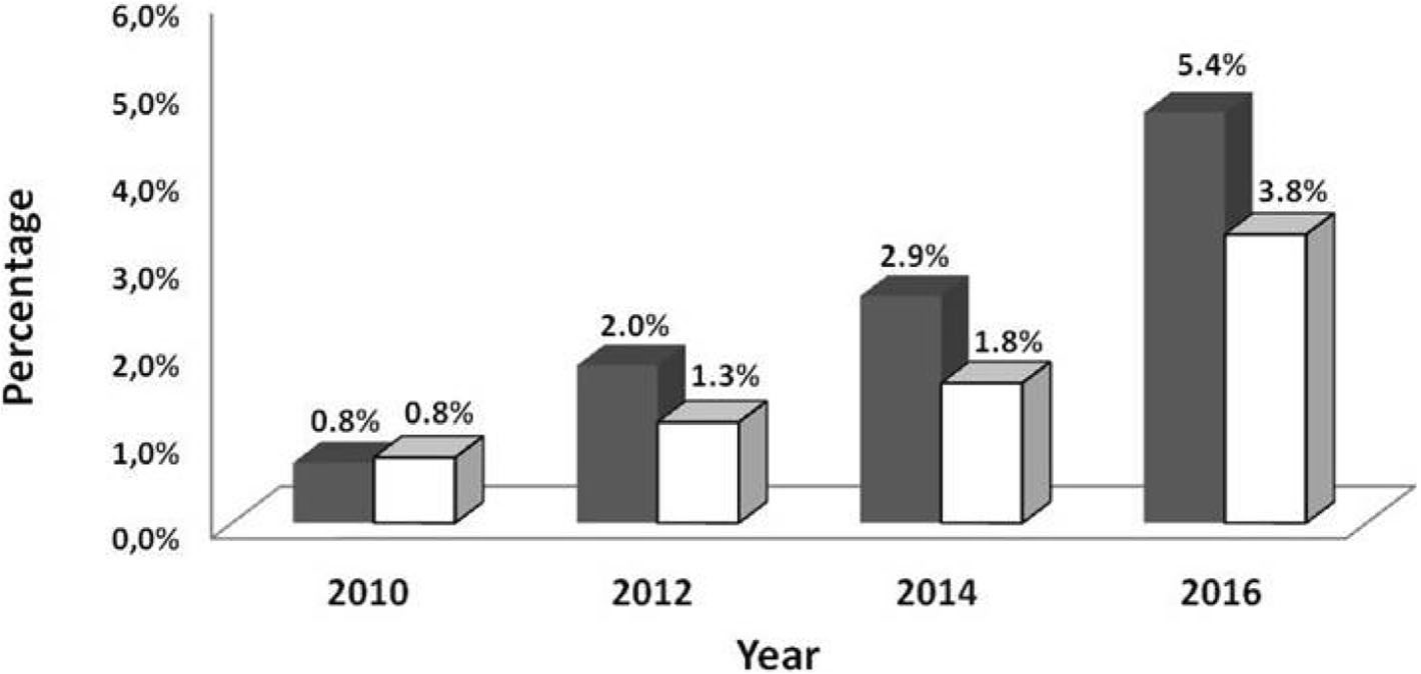
Rates of mechanical thrombectomy performed in Germany, in urban (grey) and rural (white) areas from 2010 to 2016

The temporal evolution of MT rates dependent on the population density is shown in Fig. 3. MT rates in urban (> 500 inhabitants/km^2^) and rural (< 100 inhabitants/km^2^) areas did not statistically differ in 2010(0.76% vs. 0.83%, *p* = 0.795). Thereafter, MT rates in urban settings were significantly more frequent(2012: 2.0% vs. 1.3%, p = 0·001; 2014: 2.9% vs. 1.8%, *p* < 0·001; 2016: 5.4% vs. 3.8%, *p* < 0.001.

**Fig. 4:**
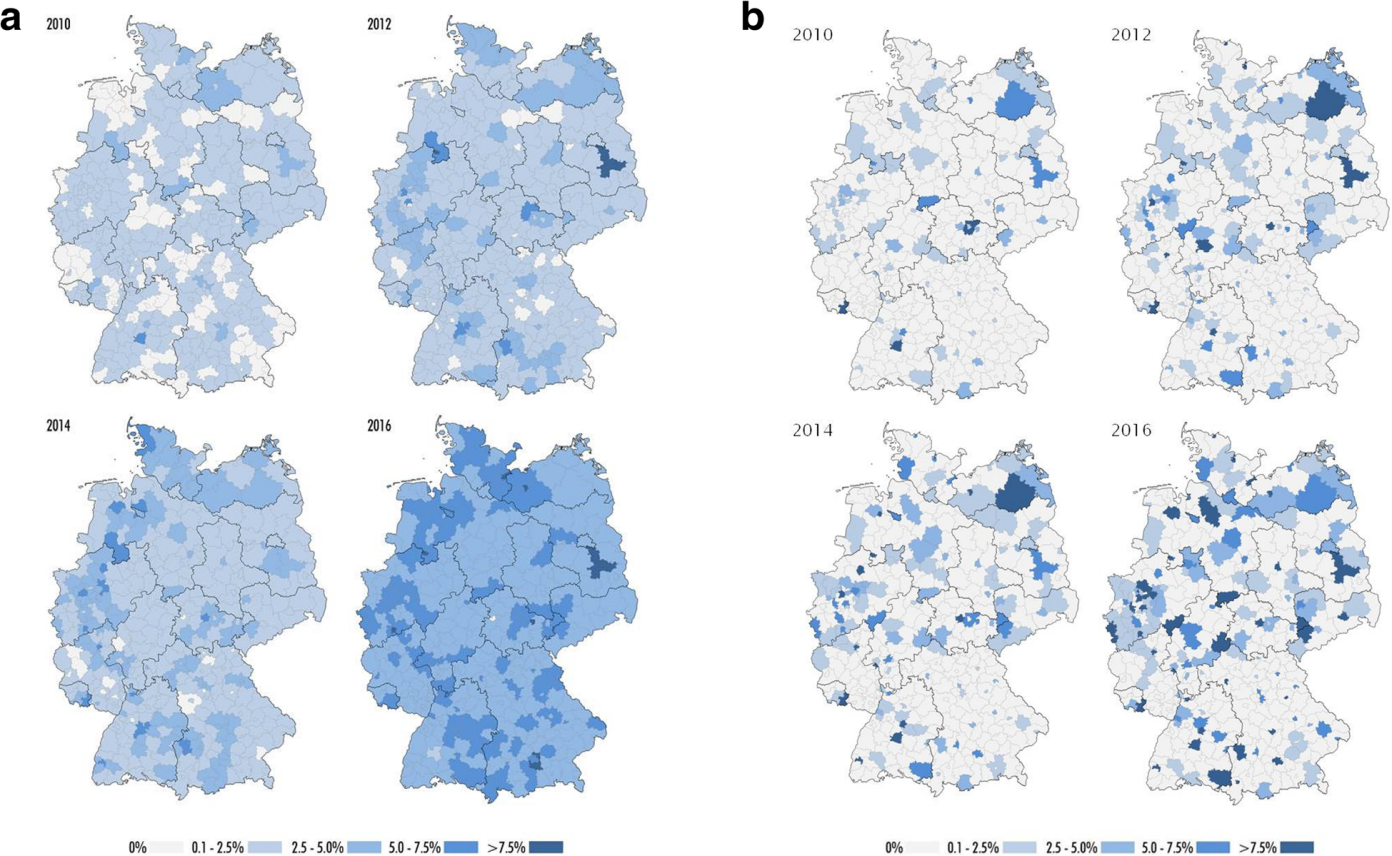
**a** Maps of temporal and regional evolution of mechanical thrombectomy rates in Germany from 2010 to 2016 according to places of residence of the patients. **b** Maps of temporal and regional evolution of mechanical thrombectomy rates in Germany from 2010 to 2016 according to places of treatment in hospital

Figure 4 depicts the temporal and regional evolution of the MT rate in all 413 German cities and districts from 2010 to 2016. Figure 4(a) illustrates the MT rates according to place of residence and Fig. 4(b) illustrates the numbers according to place of treatment.

**Fig. 5:**
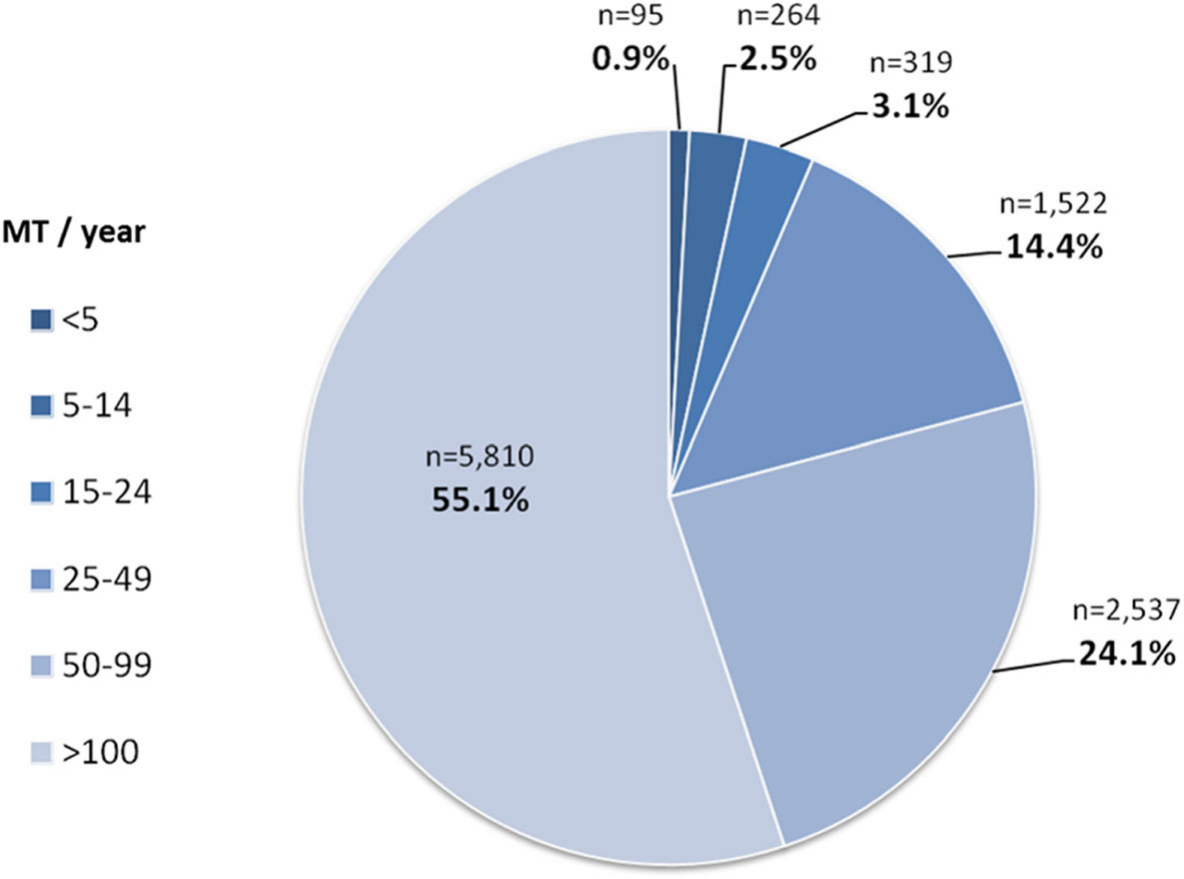
Proportion of patients being treated in different centers, classified according to the number of MT performed per year

The number of hospitals coding MT without having a neurological department according to the available structured quality reports was 16 in 2010 and 31 in 2016 with 34 procedures in 2010 and 46 procedures in 2016 accounting for 2.1 and 0.4%, respectively. These hospitals were excluded in the following results as mentioned above. Detailed results of hospitals with different MT rates can be seen in Table 2 and Fig. 5. 62% of all hospitals coding MT treated fewer than 50 procedures in 2016 accounting for 20% of all procedures. The number of centers with regular and frequent MT procedures (50–99 / 100–199 MT per year) increased considerably with procedure numbers accounting for 33% / 0% in 2010 and for 24% / 33% in 2016, respectively. There were 9 high volume neurointerventional centers (> 200 MT/year) in 2016 with a total of 2297 MT procedures performed, accounting for 22% of all MT procedures.

**Table 2:** German hospitals with a dedicated department of Neurology encoding mechanical thrombectomy (MT, OPS 8–836.80) from 2010 to 2016 categorized by numbers of MT performed per hospital and illustration of numbers of MT performed in the categories

Numbers of MT per hospital	Numbers of hospitals and procedures in 2010	Numbers of hospitals and procedures in 2012	Numbers of hospitals and procedures in 2014	Numbers of hospitals and procedures in 2016
1–9	40 /143	52 /230	55 / 182	49 /148
10–34	36 / 638	48 / 1040	49 / 992	53 / 1168
35–49	7 / 282	14 / 568	19 / 804	20 / 855
50–99	8 / 533	26 / 1768	32 / 2304	35 / 2537
100–149	0 / 0	3 / 335	5 / 597	22 / 2666
150–199	0 / 0	0 / 0	0 / 0	5 / 847
200>	0 / 0	2 / 513	2 / 558	9 / 2297
Sum	91 / 1596	145 / 4454	162 / 5437	193 / 10,518

## Discussion

This nation-wide analysis indicates a direct impact of the published randomized MT trials on the overall MT rate, with a slowing increase after the first three neutral randomized MT trails (IMS III, SYNTHESIS, MR RESCUE) in 2013, [2–4] and a prompt and strong increase after publication of the positive randomized MT trials in 2014 (MR CLEAN) [5], and 2015 (ESCAPE, EXTEND-IA, SWIFT-PRIME, REVASCAT) [6–9]. On the other hand, almost 20,000 acute stroke patients were treated with MT from 2010 till the end of 2014 without clear evidence from randomized trials. This high patient number shows the potential for recruitment into randomized controlled MT trials if reimbursement had been linked to study participation like in the Netherlands with MR CLEAN.

The mean age of patients receiving MT increased after 2015 mainly due to a higher proportion of octogenarians, almost reaching the mean age of patients treated with intravenous rt-PA alone.

Not unexpectedly, the MT rate according to place of residence varied substantially in 2016 between 0% in one small rural area and 11.2% in a larger metropolitan area. Acute ischemic stroke patients living in urban areas had a significantly higher chance to receive MT from 2012 onwards compared to patients living in rural areas. This emphasizes the need for developing regional-specific neurovascular network solutions. Currently, different approaches are debated for patients with LVO: “drip and ship” with primary transportation to the closest stroke unit, standard diagnostics, and secondary transportation to an interventional centre with bridging IVT if indicated; or “direct to mothership” with direct transportation of severely affected stroke patients to a neurointerventional centre. Patients receiving MT under the latter condition have better functional outcome mainly due to a significantly shorter delay between first brain imaging and groin puncture [17, 18] since “time is brain” is also valid in stroke patients treated with MT [19]. On the other hand, “drip and ship” reduces the time delay to standard IVT therapy and stroke unit treatment in the remaining predominant group of patients without indication for MT. A considerable amount of time (namely the time interval between admission at the interventional centre to groin puncture) could be saved in stroke patients secondarily referred for MT in the metropolitan area of the Neurovascular Net Ruhr due to prior notification about eligible patients and standardized operating procedures [17, 20]. In some settings a third approach, “trip and treat”, where the mobile interventional stroke team resorts to the primary stroke centre, may utilize the advantages of time sensitive initiation of thrombolysis with simultaneous on-site preparation for interventional therapy [21].

Our analysis of the latest available data for Germany in 2016 demonstrate that only 1% of all MT were performed in hospitals with less than 10 MT procedures per year. The number of MT procedures performed in hospitals with only occasional treatment (up to 50 MT/year) increased only moderately over the past 6 years, while centers with regular, frequent and highly frequent MT procedures increased considerably from 2010 to 2016. Overall, 80% of all MT procedures in 2016 were performed in neuro-interventional centers with at least ≥50 MT/year which is in our opinion an adequate number to provide sufficient technical and organizational expertise along with 24/7 MT service to treat directly admitted and secondarily referred stroke patients with LVO.

The three departments with the most procedures in 2016 performed 386, 362, and 234 MTs, respectively. On the basis of theoretical considerations, 5–15% of all stroke patients or 14-41 per 100.000 inhabitants^3^ can be expected to be eligible for MT in Germany [14–16]. Considering an average value of 28 per 100.000 or 10% of all strokes, the three hospitals with the most MT procedures in 2016 should therefore have a population coverage of approx. 1.38mio, 1.29mio, and 840.000 inhabitants, respectively. However, center no.1 is located in a metropolitan area in a city of approx. 583,000 inhabitants with another treatment center in the city and another 6 regular, frequent, or high volume centers in an ambit of 50 km. Center no.2 is located in a city of approx. 628,000 inhabitants with another frequent treatment center some 15 km away, and center no.3 is located in a city of 160,000 inhabitants with a city of 304.000 inhabitants only 20 km away without a regular treatment center.

These examples demonstrate the inhomogeneous infrastructure neurointerventional centers have to deal with. Three major aspects may influence strategic orientation of hospitals with (potential) interventional departments. A) local and regional patient care; B) adequate treatment rates to keep up quality standards; C) potential financial incentives and reputation. Stroke unit treatment compensation is the sound basis of stroke medicine in Germany. Within the DRG system, the compensation of a stroke patient increases by approx. 2.3 fold if MT is performed on top of systemic thrombolysis and stroke unit treatment of more than 72 h. We assume that about 13% of the absolute amount of compensation for stroke treatment (I63) in 2016 has been spent for MT compensation^4^. It stands to reason that hospital managers might not want to miss the potential financial amenities of interventional stroke treatment. Hence, these numbers also imply that financial considerations are to be considered as one incentive to offer MT. At the same time, the definitions of structural requirements to enable rapid and 24/7 access for MT for acute stroke patients with LVO as well as minimal treatment numbers for neuro-interventionalists constitute feasible instruments to foster standards of quality. To tackle this balancing act, it is the responsibility of the respective professional societies to guide infrastructural developments in order to prevent falsely driven decisions for interventional centers in the whole range of treatment frequencies. A good coverage of high quality stroke care rather than financial interests must be our number one incentive.

The decreasing percentage of bridging IVT observed over the years is most likely not primarily caused by a paradigm shift from “drip and ship” to “direct to mothership”, rather than by an increasing number of MT procedures performed without bridging-IVT in larger neuro-interventional centers and/or due to consisting contraindications for IVT, i.e. unknown time window or wake up strokes, known malignancies, recent operations, etc [22, 23]. It has to be pointed out, that direct MT without bridging IVT performed in IVT-eligible patients directly admitted to neuro-interventional centers is still a matter of debate and that prospective randomized trial addressing this question are under way (SWIFT DIRECT, MR CLEAN NO IV).

Our data refer to the overall number of a main diagnosis of ischemic stroke (ICD I63) irrespective of, e.g., symptom onset time, clinical details or basis of indication, such as vascular occlusion status or symptom severity. However, these administrative data have high quality and accuracy because registration of all ischemic stroke cases and acute treatment procedures is a prerequisite to get financial compensation, and the coding of operating and procedure keys for MT and IVT are closely controlled by medical services of the health insurances. The system itself assures that one IVT or MT procedure refers only to one acute ischemic stroke patient, even if the patient has been transferred to a second hospital for MT. Furthermore, no change in coding standards occurred in the analyzed time period. Thus, in contrast to observational registries, our data are very robust for Germany with a very low risk of missing patients, double coding of procedures, resulting in high validity and consistency.

The observed increase of patients with the main diagnosis of ischemic stroke over time can be attributed to different aspects. It is likely that patients with stroke are now more often adequately diagnosed due to the increasing number of stroke units over the years and awareness for stroke. Secondly, both first and recurrent ischemic strokes are included in our data, which might has contributed to the observed overall increase of the code I63 in an aging population. Finally, there is the possibility that reimbursement of the diagnosis ischemic stroke and of the related MT procedure resulted in a higher amount of coding over time and as a consequence in an increase of the raw ischemic stroke rate. Stroke rates from our study cannot be compared with different ICD 10 data used for causes of death in the Global Burden of Diseases Report [1].

To date, there is only scarce data on national availability of MT in acute ischemic stroke patients. Hassan and co-workers analysed the utilization of MT in the US from 2004 to 2007 (so called “post-MERCI” period) and from 2008 to 2009 (so called “post-Penumbra” period) and reported an overall endovascular treatment rate of 0.1% in 2004 and 0.6% in 2009 with a MT rate of only 0.2% in patients aged ≥85 years [24]. A more recent analysis from the National Inpatient Sample in the US reported an increase of MT use in stroke patients from 0.6% in 2008 to 1.1% in 2012 [25]. In a prospective Korean hospital based registry with up to 14 participating hospitals (mostly university hospitals in metropolitan areas) over the time period from April 2008 to November 2013, a total of 3517 of 27.851 (12.7%) stroke patients received any acute recanalization treatment, i.e. IVT and / or interventional therapy, and 1269 (36.1%) of the 3517 patients were treated with endovascular procedures alone or in combination with IVT (4.5% of all included cases) [26]. More recently, a survey from national scientific societies and stroke experts in 44 European countries estimated that 7.3% (95% CI 5.4–9.1%) of incident ischemic stroke patients received IVT in 2015 or 2016, and 1.9% (95%CI 1.3–2.5%) received MT in 2016 [27]. The estimations used in this analysis were mostly based on data from national stroke registries. An annual number of 30.000 IVT and 9.000 MT procedures were estimated for Germany, which is in a similar range compared to our administrative data [27].

To our knowledge, the hospital-based analysis presented here is the first serial nationwide coverage of actual MT treatment rates and suggest a widespread uptake of this new treatment in Germany. Population-based data is still lacking.

## Conclusion

The nation-wide rate of IVT in acute ischemic stroke in Germany has increased over the past years and an exponential increase of MT procedures was detected after publication of the positive study results. Mean age of patients undergoing MT has continuously increased and has recently almost reached the mean age of patients undergoing IVT. Therefore, MT can be regarded as an established standard therapy in some (mostly urban) regions of Germany. However, the wide range of both the IVT and MT rates in German ischemic stroke patients indicates the need for further improvement of access to acute recanalization therapies in many, mainly rural regions. Infrastructural improvement should focus both on access to neuro-interventional centers with adequate expertise, establishment of reliable standard operating procedures for transfer for MT, as well as strengthening the competence of local stroke units for standard care including IVT and rapid and reliable recognition of MT candidates with standardized brain and vessel imaging.

There is a trend towards medium and high volume treatment centers in Germany in 2016 with most MT procedures (80%) performed in centers with ≥50 MT procedures per year in 2016. It is expected in the near future that stroke societies will recommend that at least 35 to 50 MT procedures should be performed in a neuro-interventional center per year for quality reasons.

## Abbreviations

DRG: Diagnosis Related Groups
ICD: International Classification of Diseases
IVT: intravenous thrombolysis
LVO: large vessel occusion
MT: mechanical thrombectomy
OPS: Operating and Procedure Key
rt-PA: recombinant tissue plasminogen activator

## Footnotes

1 For financial compensation in Germany, all in-hospital cases are encoded by International Statistical Classification of Diseases and Related Health Problems 10th revision, German modification (ICD-10-GM) and relevant operating and procedure keys (OPS codes). In the analyzed time period there was no change in the German coding system or a revision of the ICD version. Data analyzed in this manuscript are publicly accessible in general. However, the *original* data of the hospitals’ quality reports subject to §137 Abs. 3, 1 No. 4 SGB V are not directly accessible. In order to analyze the data complexity as done in this manuscript, the whole data base has to be obtained as *extensible markup-language-version* and then to be readouted by a specific program. A complete and unaltered subscription of the quality reports is available with the *Gemeinsamer Bundesausschuss* (G-BA, Federal Joint Committee): https://www.g-ba.de/institution/themenschwerpunkte/qualitaetssicherung/qualitaetsdaten/qualitaetsbericht/xml-daten/). Likewise, DRG raw data by *Destatis* in the data complexity analyzed here are not publicly visible and have to be obtained as paid service for users as either *zip-* or *access*-data file (https://www.destatis.de/DE/Service/Kontakt/Kontakt.html).

2 Structured Quality Reports of the hospitals according to §137 3.1 No. 4 SGB V (reported years 2010–2016), XML-version, G-BA

3 Assuming a stroke incidence of 276 per 100.000 with 227.687 strokes (ICD I63) in Germany in 2016 (see above) and a population of 82.500.000 inhabitants (https://www.destatis.de/DE/PresseService/Presse/Pressemitteilungen/2018/01/PD18_019_12411.html)

4 Even though difficult to calculate for methodological reasons, we calculated real costs of about 1.6 bn Euro for all coded I63 cases with about 211 mio for all cases comprising MT (OPS 8–836.80).
